# *In silico* study of potential anti-SARS cell entry phytoligands from *Phlomis aurea*: a promising avenue for prophylaxis

**DOI:** 10.2217/fvl-2021-0031

**Published:** 2021-10-28

**Authors:** Amira R Khattab, Mohamed Teleb, Mohamed S Kamel

**Affiliations:** 1 ^1^Pharmacognosy Department, College of Pharmacy, Arab Academy for Science, Technology & Maritime Transport, Alexandria, 1029, Egypt; 2 ^2^Department of Pharmaceutical Chemistry, Faculty of Pharmacy, Alexandria University, Alexandria, 21521, Egypt; 3 ^3^Pharmacognosy Department, Faculty of Pharmacy, Deraya University, Minia, 61111, Egypt; 4 ^4^Pharmacognosy Department, Faculty of Pharmacy, Minia University, Minia, 61519, Egypt

**Keywords:** ACE2, molecular docking, *Phlomis aurea*, SARS-CoV-2, spike protein

## Abstract

**Aim:** The severity of COVID-19 has raised a great public health concern evoking an urgency for developing multitargeted therapeutics. *Phlomis* species was ethno-pharmacologically practiced for respiratory ailments. **Materials & methods:** An array of 15 phytoligands previously isolated from *Phlomis aurea* were subjected to molecular docking to explore their potential SARS-CoV-Spike-angiotensin-converting enzyme 2 complex inhibition, that is essential for virus entry to host cell. **Results:** Acteoside (**11**) showed the most potent *in silico* inhibition with an additional merit, over hesperidin (**16**), of not binding to angiotensin-converting enzyme 2 with well proven *in vivo* pulmonary protective role in acute lung injury, followed by chrysoeriol-7-*O*-β-glucopyranoside (**12**) and luteolin-7-*O*-β-glucopyranoside (**14**). **Conclusion:** Phytoligands (**11**, **12** and **14**) were posed as promising candidates with potential prophylactic action against COVID-19. These phytoligands were prioritized for further biological experimentation because of their acceptable predicted ADME and drug-likeness parameters. Moreover, they could aid in developing multitargeted strategy for better management of COVID-19 using phytomedicines.

SARS-CoV-2, an enveloped-virus possessing single-stranded, positive-sense RNA genome, is the causative agent of the rapidly evolving novel COVID-19 which raises an immense public health concern worldwide. The SARS-CoV membrane comprises of four structural proteins *viz.* S (spike), E (envelope) and M (membrane) proteins that create viral envelope and N (nucleocapsid) proteins that hold the viral RNA genome. These proteins are responsible for the critical step in viral infection which is the viral entry to the host cells. SARS-CoV invades human cells through the main envelope embedded spike (S) glycoprotein which contains a receptor binding domain. Receptor binding domain then binds to the metallopeptidase on host cells, angiotensin converting enzyme-2 (ACE-2) [1]. The structural investigation of SARS-CoV-Spike-ACE2 complex and the viral entry mechanism aided in better understanding the disease pathogenesis and developing vaccines to prevent SARS-CoV-2 infection as well as therapeutic drugs [2]. As of today, no specific clinically proven antiviral drugs available for COVID-19 treatment; hence, computational efforts are still ongoing for the *in silico* exploration of promising drug candidates [3].

On the other side, the development of an effective SARS-CoV-2 vaccine is essential to provide an effective and economical way to prevent the viral infection and stop its transmission. These vaccines are mostly made with inactivated and attenuated viral protein particles, viral vectors and viral DNA/RNAs. Unfortunately, vaccine preclinical and clinical trials and production typically require a lengthy process which is additionally challenged by the high mutation rate of SARS-CoV-2 (about 25 mutations per year) and the diversified antigenic sequence of SARS-CoV-2 virus [4]. Accordingly, the findings of the *in silico* studies are considered a magic bullet for medicinal chemists to accelerate the development of preventive and therapeutic drugs for the virulent SARS-CoV-2 viral infection. Such drugs can provide valuable preventive and therapeutic intervention until an effective vaccine is produced [5].

Several research endeavors are still ongoing to find drug leads that can target host receptor or viral proteins for COVID-19 management [6–9]. Natural therapeutic agents that can block the first step of viral infection, in other words, viral entry, by preventing its binding with ACE2, are considered an important class of antiviral therapeutics that can also be of prophylactic potential. Only few S-protein-ACE2 binding inhibitors have been reported up till now, among which tetra-*O*-galloyl-β-D-glucose, emodin and hesperidin [1,10].

*Phlomis* species (Lamiaceae family), is one of the endemic plants to Saint Katharine mountain (southern Sinai, Egypt) that was described by Dioscorides as herbal medicine and ethno-pharmacologically practiced for respiratory ailment treatment [11]. Besides, many *Phlomis* species were used in Anatolian folk medicine as an antimicrobial, antimalarial, immunosuppressive and as a stimulant [12]. *Phlomis* species are also well reported to exhibit anti-infective activities, reduce fever, treat cough and throat infections among which *P. caucasica* Rech. F and *P. cephalotes* Roth (malarial fever) [13]. However, a very limited number of reports exists on the antiviral potential of *Phlomis* species, except for *P. pungens* var. pungens against parainfluenza (PI-3) RNA virus [14]. A wide array of phytochemical classes was identified in *Phlomis* genus *viz.* iridoids, lignans, flavonoids, alkaloids, phenylpropanoids, monoterpenes, diterpenoids and their glycosides [15].

Our strategy in the discovery of natural anti-SARS-CoV-2 therapeutics is contemplated by searching for drug candidates that can block the critical early step in viral infection *viz.* viral cell entry. Accordingly, we performed docking simulation for an array of 15 phytoligands, previously isolated from *Phlomis aurea* Decne (Syn.: *P. flavescens* Miller and *P. angustifolia* Miller) (Figure 1) [15], in order to predict their relative antiviral potential to interrupt the interaction between human ACE2 receptor and viral spike protein. Further, *in silico* prediction of physicochemical properties, absorption, distribution, metabolism, excretion and toxicity (ADMET) and drug-likeness parameters of *Phlomis* phytoligands were achieved.

**Figure 1.**
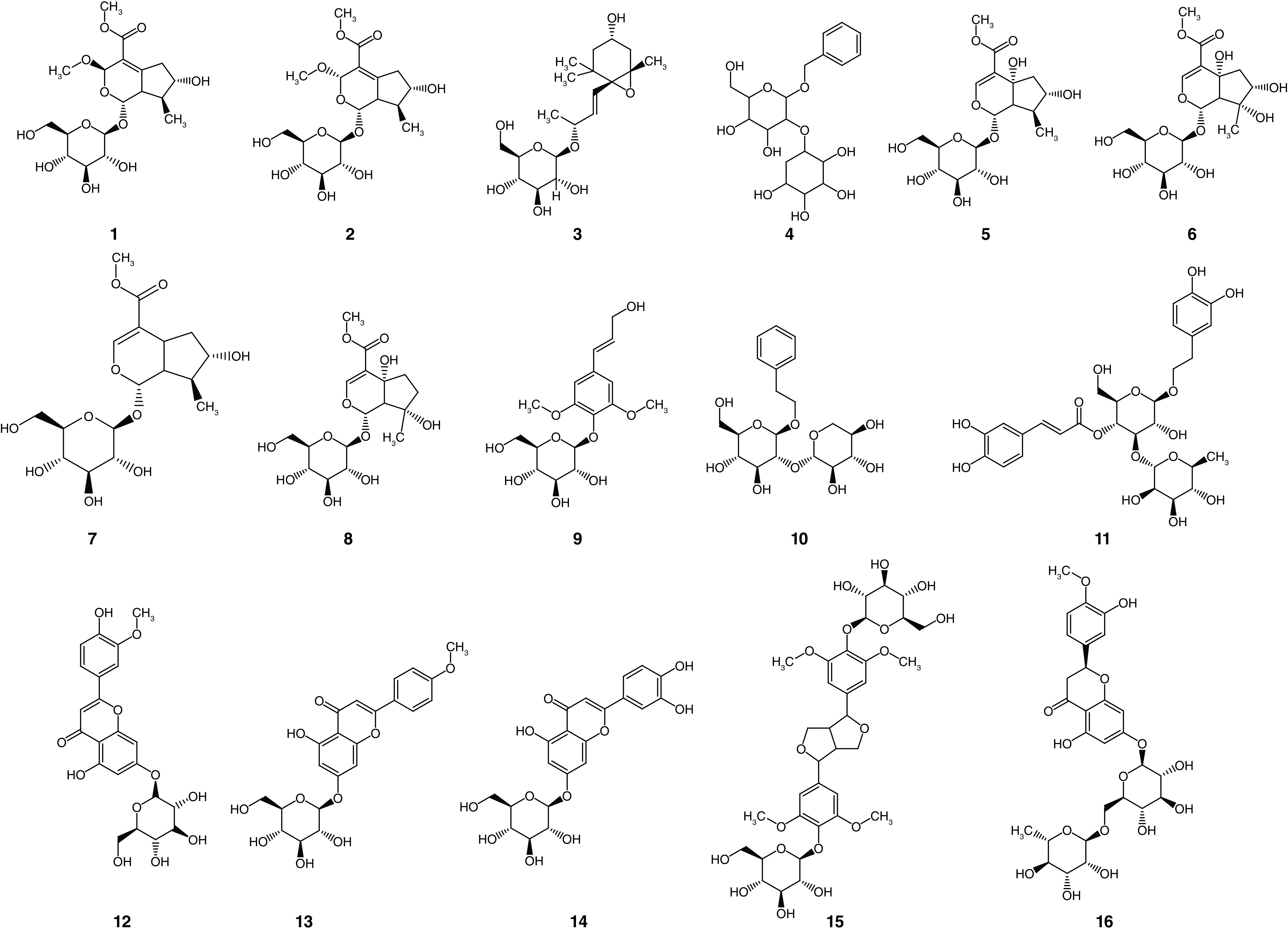
Chemical structures of the studied phytoligands from *Phlomis aurea*. (**1**) 3-epiphlomurin. (**2**) Phlomurin. (**3**) Phlomuroside. (**4**) Benzyl alcohol-*O*-β-xylopyranosyl-(1→2)-β-glucopyranoside. (**5**) Auroside. (**6**) Lamiide. (**7**) 8-epiloganin. (**8**) Ipolamiide. (**9**) Syringin. (**10**) 2-phenylethyl-*O*-β-xylopyranosyl-(1→2)-β-glucopyranoside. (**11**) Acteoside. (**12**) Chrysoeriol-7-*O*-β-glucopyranoside. (**13**) Acacetin-7-*O*-β-glucopyranoside. (**14**) Luteolin-7-*O*-β-glucopyranoside. (**15**) Liriodendrin. (**16**) Hesperidin.

## Materials & methods

### Phytoligands under study

*In silico* exploration was performed on an array of 15 phytoligands (Figure 1) previously isolated from leaves of *Phlomis aurea* Decne from Saint Katharine mountain (Sinai, Egypt) which belonged to different phytochemical classes *viz.* iridoids, in other words, 3-epiphlomurin (**1**), phlomurin (**2**), auroside (**5**), lamiide (**6**), 8-epiloganin (**7**) and ipolamiide (**8**), megastigmane glucoside, in other words, phlomuroside (**3**), benzyl alcohol glycoside, in other words, benzyl alcohol-*O*-β-xylopyranosyl-(1→2)-β-glucopyranoside (**4**), phenolic glycosides, in other words, syringin (**9**) and acteoside (**11**), phenylethanoid glycoside, in other words, 2-phenylethyl-*O*-β-xylopyranosyl-(1→2)-β-glucopyranoside (**10**), flavonoids, in other words, chrysoeriol-7-*O*-β-glucopyranoside (**12**), acacetin-7-*O*-β-glucopyranoside (**13**) and luteolin-7-*O*-β-glucopyranoside (**14**) and lignin, in other words, liriodendrin (**15**) [15]. 2D structures of the phytoligands (**1**–**15**) were downloaded as structure-data file from PubChem (https://pubchem.ncbi.nlm.nih.gov).

### Docking simulation

Molecular operating environment (MOE) software (version 2015.10, Chemical Computing Group, QC, Canada) was employed for docking simulations using the crystal structure of SARS-CoV-2 spike (S) protein C-terminal domain (SARS-CoV-2-CTD) in complex with human ACE2 (hACE2). SARS-CoV-Spike-ACE2 complex reported to reveal the most recently known hACE2-binding mode was retrieved from the protein data bank (PDB ID: 6LZG) [16]. The complex was prepared by eliminating the unwanted residues, solvents and ligands using the default settings of the ‘structure preparation’ module. The spatial arrangement of some key residues in the SARS-CoV-2-CTD binding interface was located as the receptor site utilizing ‘site finder’ feature of MOE, where the site number 8 was selected. Structures of the phytoligands (**1**–**15**) under investigation were built *in silico*, then subjected to default energy minimization and geometry optimization. The ligand placement was set to apply triangular matcher algorithm. Top five conformers of the test ligands that possessed nonredundant poses of the lowest binding energy were generated by utilizing alpha HB as the default scoring function. An induced fitting protocol for docking was employed to record the best possible molecular interactions [17]. Phytoligands were then ordered according to their S-scores with a root-mean-square deviation (RMSD) value <2 Å in addition to the graphical representations of the ligand interactions.

### *In silico* prediction of physicochemical properties, ADMET & drug-likeness parameters

**Figure 2.**
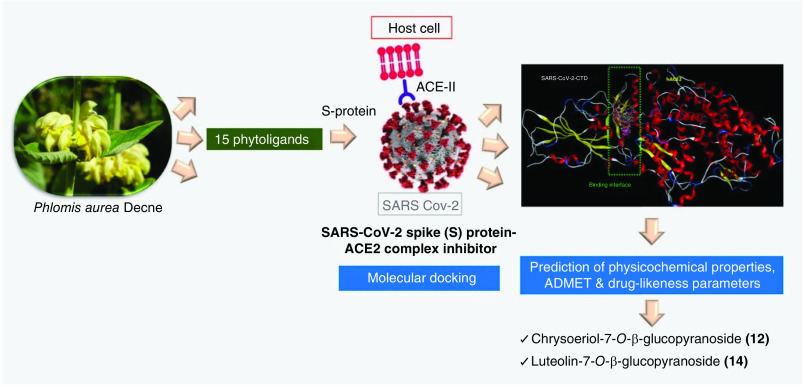
A workflow of the research methodology.

Physicochemical properties and drug-likeness were computed by SwissADME software [18]. ADME profiling was performed by PreADMET calculator [19]. The workflow of the research methodology is illustrated in Figure 2.

## Results

### Molecular docking analysis of *Phlomis aurea* phytoligands

Viral infections are generally initiated with the binding of viral particles to specific host-cell surface receptors. Hence, receptor recognition is considered as a critical determinant of the viral entry and cell invasion. Targeting such process is; therefore, considered an inspiring prophylactic approach against viral infection. In SARS-CoV-2, the viral entry is mediated by an envelope-embedded surface-located spike (S) glycoprotein [20] which utilizes hACE2 for cell entry [21]. The S glycoprotein is cleaved, in most cases, by host cell proteases into S1 and S2 subunits for receptor recognition and cell membrane fusion, respectively. S1 is further subdivided into N-terminal domain and C-terminal domain (CTD), both can function as a receptor-binding entity. Most recently, a pioneer study utilized immunostaining and flow cytometry assays to first identify the S1 CTD of SARS-CoV-2 as the key region that interacts with the hACE2 receptor. A 2.5-A° crystal structure of SARS-CoV-2-CTD in complex with a single hACE2 molecule in asymmetric unit (PDB ID: 6LZG) [16] was solved revealing a clear receptor-binding mode. Further analysis of the virus–receptor interaction on structural basis was performed to identify the key aminoacids involved in complex formation. Accordingly, a series of hydrophilic residues forming an H-bonds network and salt bridge interactions were located along the binding interface [16].

In light of the mentioned information we utilized docking simulation to probe the ability of the studied phytoligands to destabilize the virus–receptor complex or even prevent its formation, where possible accommodation/fitting of such phytochemicals at the SARS-CoV-2-CTD-h2ACE interface and their interaction with the complex key amino acids may provide preliminary promising insights for further biological investigation. Several *in silico* studies displayed the possible inhibitory mechanisms of various phytochemicals on the ACE2–Spike complex of SARS-CoV-2, and highlighted the structural determinants of important interactions [22–26]. In absence of a cocrystalized inhibitor at the interface of the studied complex, we employed the ‘site finder’ feature of MOE 2015.10 to locate the most suitable site for docking the studied phytochemicals into the SARS-CoV-2-CTD-2hACE binding interface taking in consideration the key residues involved in the complex formation. Flexible docking of the 15 phytochemicals under investigation was performed several times in the defined site at the binding interface with hesperidin as a reference viral entry inhibitor [27].

Docking simulations results (Table 1) showed that most of the studied phytoligands displayed good binding affinities compared with hesperidin (**16**). Acteoside (**11**) came at the top of the list recording the best binding affinity (-7.75 kcal/mol) among the studied phytoligands, even better than hesperidin (-7.10 kcal/mol).

**Table 1.** Docking simulations results of the studied phytoligands (1–15) from *Phlomis aurea*.

n	Name of phytoligands	ΔG^†^ (kcal/mol)	Interactions at the binding interface^‡^	
			hACE2 residues	SARSCoV-2-CTD residues
**1**	3-Epiphlomurin	-4.70	Not studied	Not studied
**2**	Phlomurin	-6.65	Glu37	Arg403, Arg408
**3**	Phlomuroside	-6.07	Glu37	Glu406, Arg408
**4**	Benzyl alcohol-*O*-β-xylopyranosyl-(1→2)-β glucopyranoside	-6.23	Glu37, **Lys353**	**Tyr453**, **Gly496**, Tyr505
**5**	Auroside	-3.73	Not studied	Not studied
**6**	Lamiide	-5.72	**His34**, Glu37	Glu406, **Tyr453**
**7**	8-Epiloganin	-3.83	Not studied	Not studied
**8**	Ipolamiide	-5.68	No interactions	No interactions
**9**	Syringin (eleutheroside B)	-6.40	No interactions	No interactions
**10**	2-Phenylethyl-*O*-β-xylopyranosyl-(1→2)-β-glucopyranoside	-6.50	No interactions	Glu406, Gln409
**11**	Acteoside (verbascoside)	-7.75	No interactions	Glu406, Arg408, Gln493, Ser494, **Gly496**
**12**	Chrysoeriol-7-*O*-β glucopyranoside	-6.72	No interactions	**Tyr453**, **Gly496**
**13**	Acacetin-7-*O*-β-glucopyranoside	-6.06	**His34**, **Lys353**	**Tyr453**, Ser494, **Gly496**
**14**	Luteolin-7-*O*-β-glucopyranoside	-6.73	**His34**, **Lys353**	**Tyr453**, Ser494, **Gly496**
**15**	Liriodendrin (syringaresinol-di-*O*-glucoside)	-5.97	No interactions	Arg403, Glu406, Arg408 Gln409, **Lys417**
**16**	Hesperidin	-7.10	**His34**, Ala387	Gln409, **Lys417**, Ser494

† The best ligand–receptor complex binding free energy at RMSD <2 Å.

‡ The key residues involved in the SARS-CoV-2-CTD-2hACE complex formation are listed in bold.

Liriodendrin (**15**), acacetin-7-*O*-β-glucopyranoside (**12**), phlomurin (**2**), 2-phenylethyl-*O*-β-xylopyranosyl-(1→2)-β-glucopyranoside (**10**), Syringin (**9**), benzyl alcohol-*O*-β-xylopyranosyl-(1→2)-β glucopyranoside (**4**), phlomuroside (**3**) and luteolin-7-*O*-β-glucopyranoside (**13**) showed slightly less binding affinities ranging from -6.73 to -6.06 kcal/mol. Moderate fitting was observed in case of the three phytoligands *viz.* chrysoeriol-7-*O*-β glucopyranoside (**15**), lamiide (**6**) and ipolamiide (**8**) with binding affinities of -5.97, -5.72 and -5.68 kcal/mol, respectively. The remaining phytoligands showed low binding affinities.

### Interaction of *Phlomis* phytoligands with human SARS-CoV-Spike-ACE2 complex

**Figure 3.**
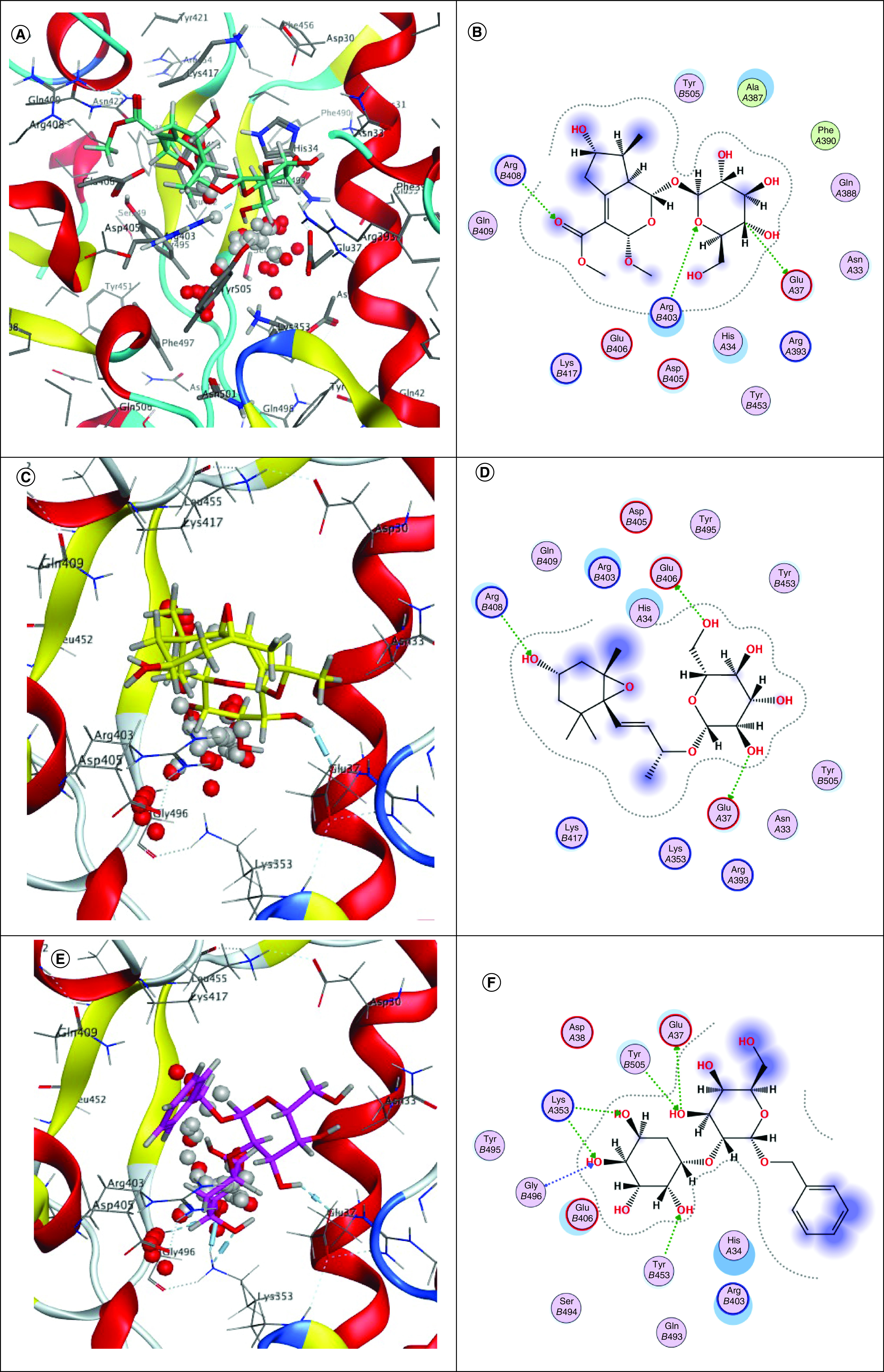

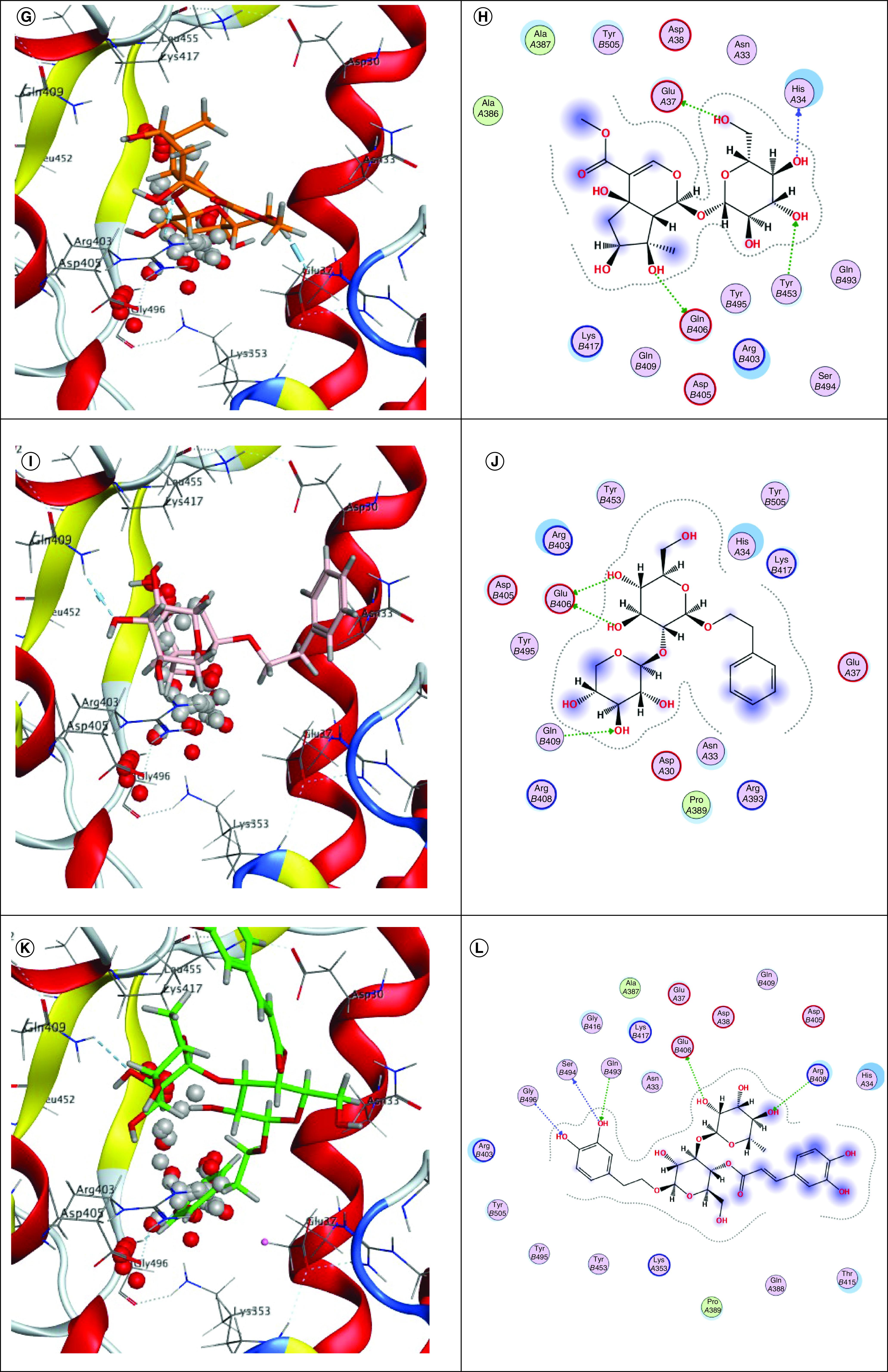

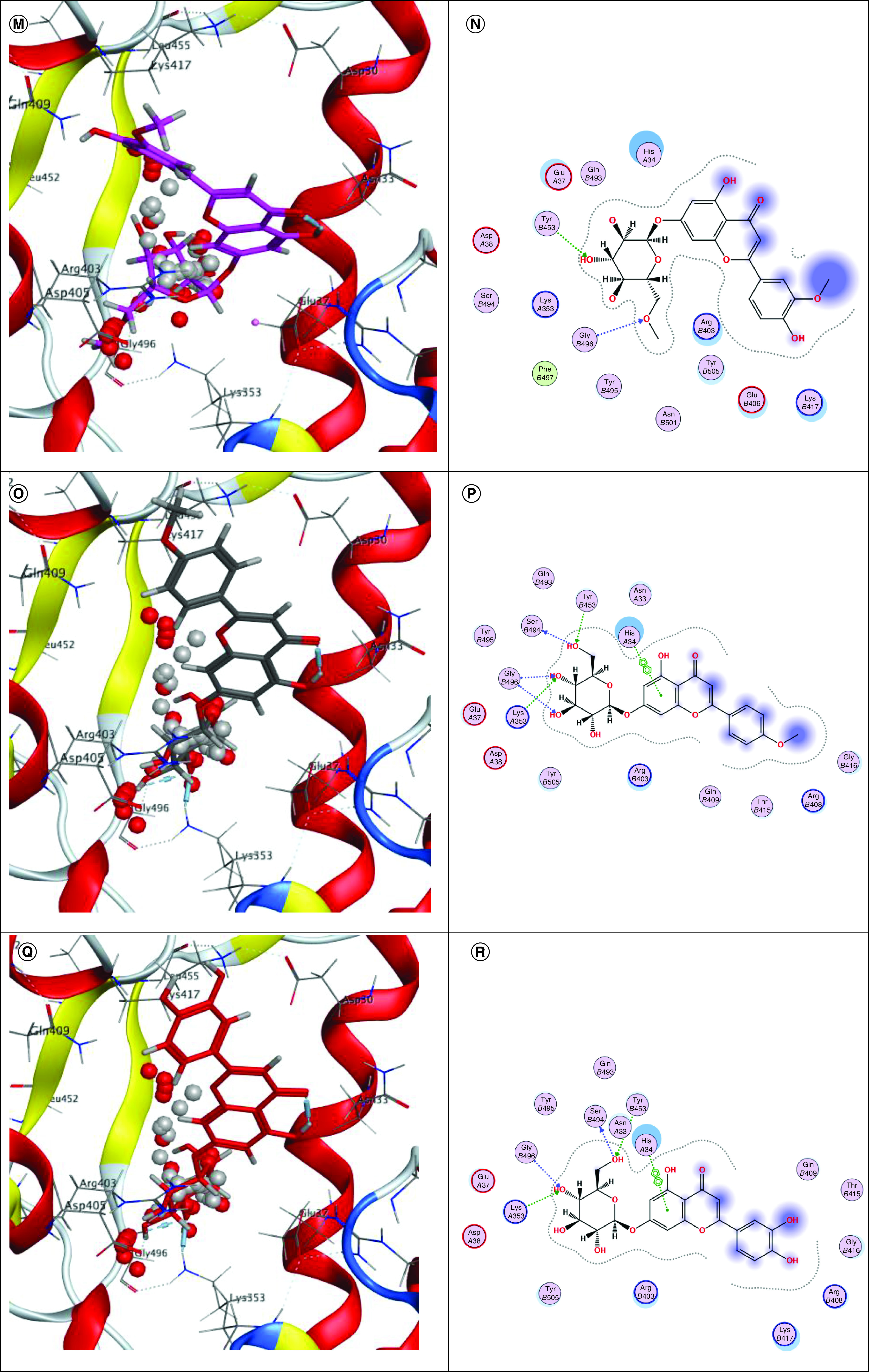

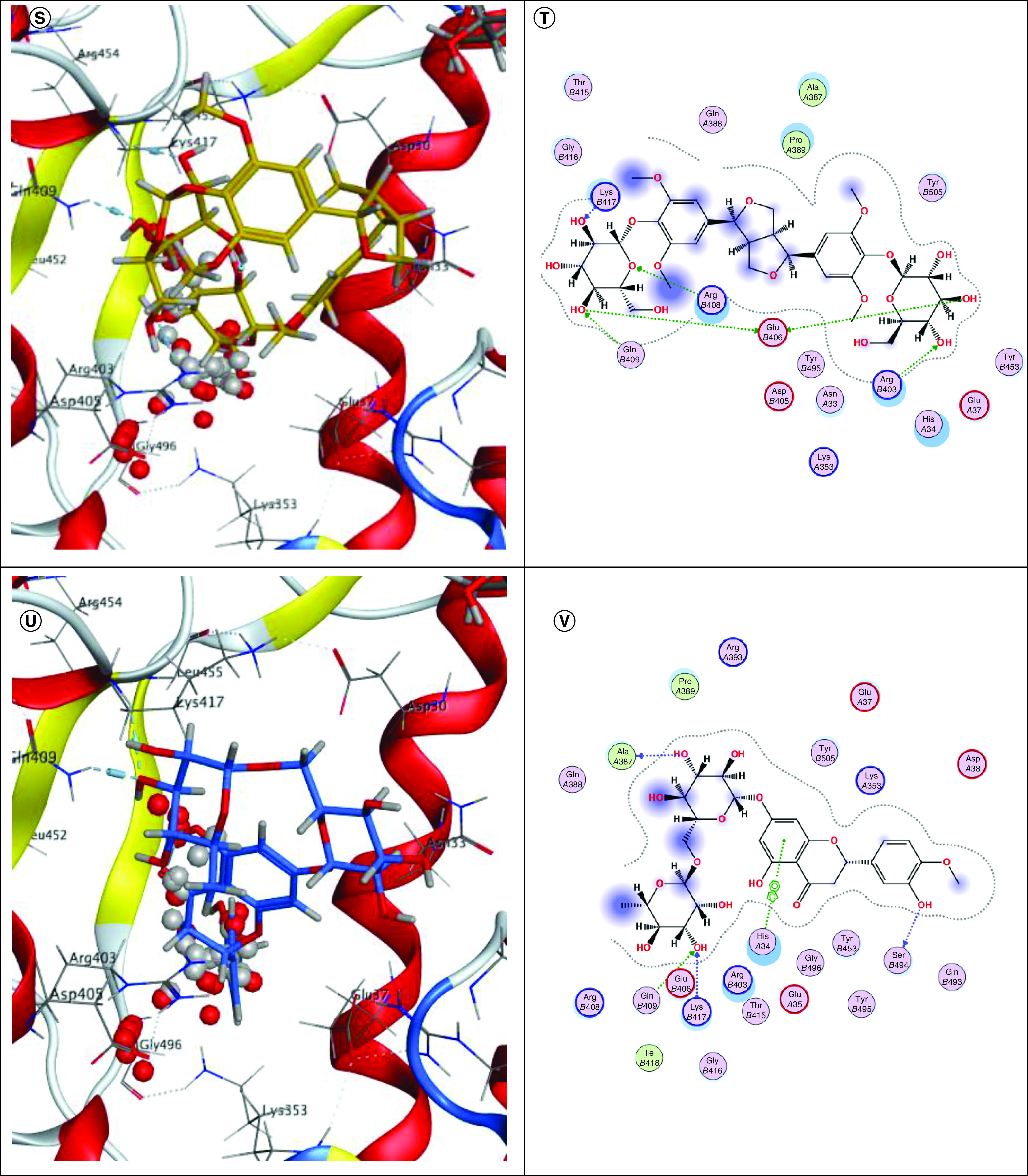
Docking simulations of the studied phytochemicals into the binding interface of SARS-CoV-2-CTD in complex with hACE2 (PDB ID: 6LZG). **(A & B)** 3D and 2D binding modes of **2** (cyan sticks). **(C & D)** 3D and 2D binding modes of **3** (yellow sticks). **(E & F)** 3D and 2D binding modes of **4** (magenta sticks). **(G & H)** 3D and 2D binding modes of **6** (orange sticks). **(I & J)** 3D and 2D binding modes of **10** (pink sticks). **(K & L)** 3D and 2D binding modes of **11** (green sticks). **(M & N)** 3D and 2D binding modes of **12** (deep pink sticks). **(O & P)** 3D and 2D binding modes of **13** (white sticks). **(Q & R)** 3D and 2D binding modes of **14** (red sticks). **(S & T)** 3D and 2D binding modes of **15** (deep yellow sticks). **(U & V)** 3D and 2D binding modes of **16** (blue sticks) in the binding interface of SARS-CoV-2-CTD in complex with hACE2 (PDB ID: 6LZG). The names of phytoligands (**1**–**15**) are given in experimental section.

**Figure 4.**
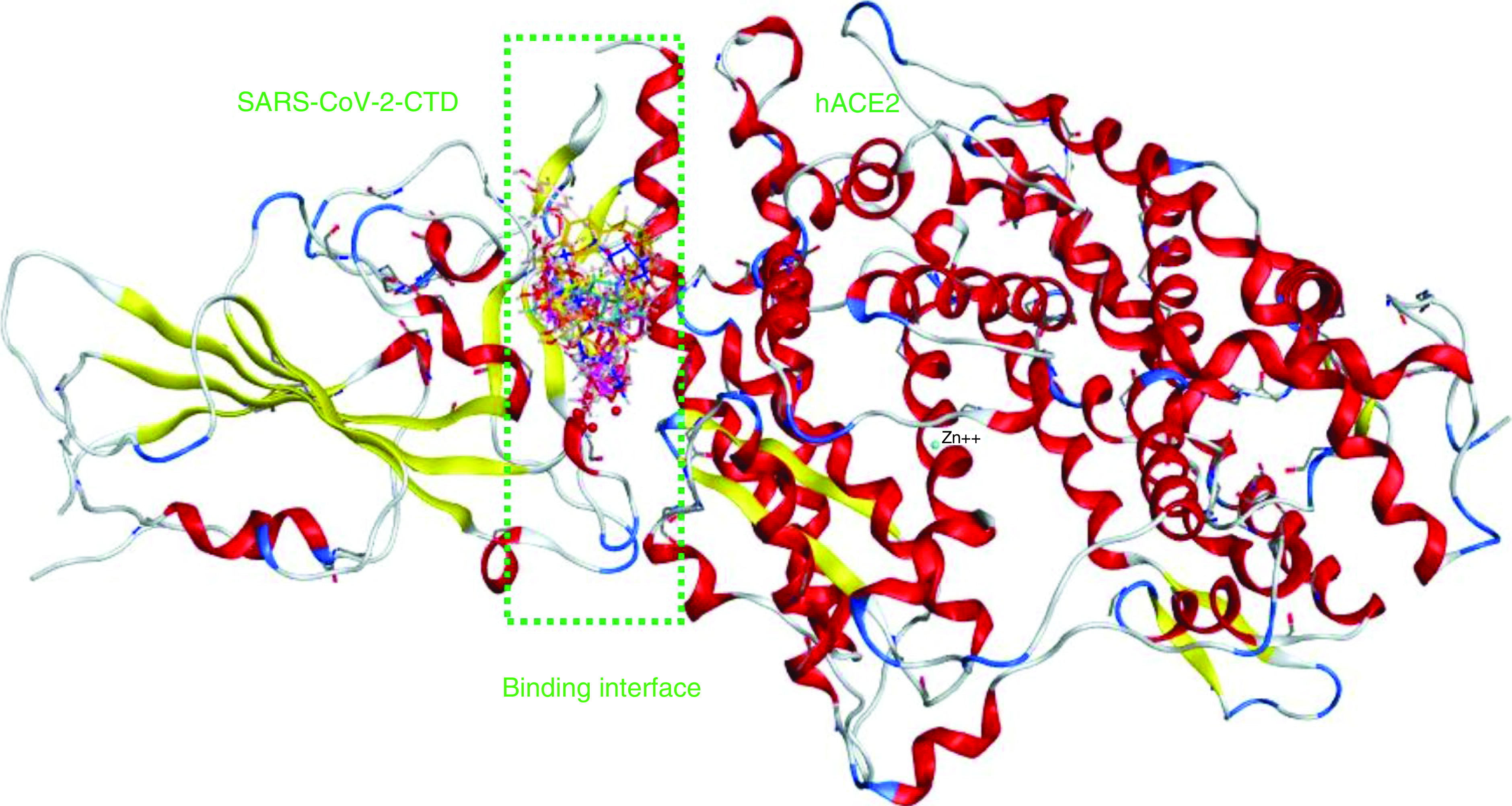
The binding interface of SARS-CoV-2-CTD in complex with hACE2 (PDB ID: 6LZG) showing an overlay of some of the studied phytoligands. *viz.* 2 (cyan sticks), **3** (yellow sticks), **4** (magenta sticks), **6** (orange sticks), **10** (pink sticks), **11** (green sticks), **12** (deep pink sticks), **13** (white sticks), **14** (red sticks), **15** (deep yellow sticks) and **16** (blue sticks). The names of phytoligands (**1**–**15**) are given in experimental section.

Some of the studied phytoligands showed promising *in silico* results upon inspecting their binding modes with human SARS-CoV-Spike-ACE2 complex. They were able to efficiently accommodate into the interface and interact with the key aminoacids mostly via hydrogen bonding (Figures 3 & 4). Hence, they can destabilize the virus–receptor complexation which is dominated by polar bonding interactions with key hydrophilic amino acid residues [16]. Acteoside (**11**), a phenolic glycoside recording the best binding affinity in the current study, exhibited hydrogen bonding interactions with Gly496 of the SARS-CoV-2-CTD engaged in complex formation (Figure 3K & L). Additionally, it displayed hydrogen bonding with the nearby amino acids, in other words, Glu406, Arg408, Gln493 and Ser494 on the SARS-CoV-2-CTD side of the interface. These interactions were posed by the sugar hydroxyl groups for Glu406 and Arg408; whereas, Gln493 and Ser494 interacted with the phenolic hydroxyl groups. This highlights that sugar hydroxyl groups and the phenolic hydroxyl groups are the important structural features of the phytoligand. Obviously, acteoside (**11**) did not show any interactions with hACE2 residues of SARS-CoV-Spike-ACE2 complex as compared with the reference hesperidin (**16**) which displayed π–π and hydrogen bonding interactions with hACE2 His34 and Ala387, respectively, in addition to its binding to SARS-CoV-2-CTD Gln409, Lys417, Ser494 (Figure 3U & V).

#### *In silico* prediction of physicochemical properties, ADMET & drug-likeness parameters of the most promising phytoligands

Recently, *in silico* prediction studies of physicochemical properties, ADMET and drug-likeness parameters are utilized for the identification of the most promising leads. Herein, *SwissADME* software was employed to compute the physicochemical properties formulating drug-likeness parameters of the hit phytochemicals (Table 2) [18,28].

**Table 2.** *In silico* predicted physicochemical properties, ADMET and drug-likeness parameters of the promising phytoligands (11, 12 & 14).

n	Physicochemical parameters							ADMET								Drug-likeness	
	Log P^†^	M.Wt^‡^	HBA^§^	HBD^¶^	NROTB^#^	TPSA^††^	S^‡‡^	HIA^§§^	PPB^¶¶^	BBB^##^	Caco-2^†††^	MDCK^‡‡‡^	CYP3A4 inhibitor	CYP2D6 inhibitor	LD_50_^§§§^	Lipiniski^¶¶¶^	Veber^###^
**11**	-2.37	624.59	15	9	11	245.29	290.5	7.67	64.28	0.031	11.10	0.045	yes	None	5000	3 violations (M.Wt, HBA, HBD)	2 violations (TPSA, rotors)
**12**	-1.89	462.40	11	6	5	179.28	40.22	42.14	62.67	0.034	5.02	0.317	yes	None	5000	2 violations (HBA, HBD)	1 violation (TPSA)
**14**	-2.10	448.38	11	7	4	190.28	85.91	25.16	73.27	0.033	4.87	0.756	yes	None	5000	2 violations (HBA, HBD)	1 violation (TPSA)

† Log P: Logarithm of compound partition coefficient between n-octanol and water.

‡ M.Wt: Molecular weight.

§ HBA: Number of hydrogen bond acceptors.

¶ HBD: Number of hydrogen bond donors.

# NROTB: Number of rotatable bonds.

†† TPSA: Polar surface area. Drug-like TPSA <140–150 A^2^.

‡‡ S: Aqueous solubility (mg/l).

§§ HIA: Human intestinal absorption. HIA values <20% (poorly absorbed), values ≈ 20–70% (moderately absorbed) and values >70% (well absorbed) [29].

¶¶ PPB: Plasma protein binding. PPB values <90% (poorly bound) and values >90% (strongly bound) [30].

## BBB: Blood–brain barrier penetration. BBB values <0.1 (low CNS absorption), values ≈ 0.1–2 (medium CNS absorption) and values >2 (high CNS absorption) [31].

††† Caco-2: Permeability through cells derived from human colon adenocarcinoma. P_Caco-2_ values <4 nm/s (low permeability), values ≈ 4–70 nm/s (medium permeability) and values >70 nm/s (high permeability) [32,33].

‡‡‡ MDCK: Permeability through Madin–Darby Canin kidney cells. P_MDCK_ values <25 nm/s (low permeability), values ≈ 25–500 nm/s (medium permeability) and values >500 nm/s (high permeability) [33,34].

§§§ LD_50_: The median lethal dose (mg/Kg). Toxicity classes according to GHS are: Class I: fatal if swallowed (LD50 ≤5), Class II: fatal if swallowed (5 <LD50 ≤50), Class III: toxic if swallowed (50 <LD50 ≤300), Class IV: harmful if swallowed (300 <LD50 ≤2000), Class V: may be harmful if swallowed (2000 <LD50 ≤5000) and Class VI: nontoxic (LD50 >5000) calculated using the ProToxII (http://tox.charite.de/) web server [35].

¶¶¶ Lipinski rule: Log p ≤ 5, M.Wt ≤500 Da, HBA ≤10 and HBD ≤5 [36].

### Veber rule: NROTB ≤10 and TPSA ≤140 [37].

## Discussion

As revealed form our results, acteoside (**11**) did not inhibit ACE2, which is an added value to its promising anti-SARS-CoV2 potential as the ACE2 inhibition is unfavorable to COVID-19 patients with already developed symptoms. These symptoms develop as a consequence to the decreased production of angiotensin 1–7, which exhibits antifibrotic, anti-inflammatory, vasodilatory actions via Mas receptor [38]. Furthermore, there are preliminary data proving that patients taking angiotensin-II inhibitors (ACE-I) exhibit severe symptoms with a higher mortality rate as compared with their counterparts not taking these medications [39]. Accordingly, ACE2 plays a protective role in the animal models of acute respiratory distress syndrome and acute lung injury [40].

Acteoside (**11**) occurs frequently in several botanical families *viz.* Scrophulariacea, Lamiacea and Verbenacea [41]. It was reported to exhibit a strong *in vivo* antiviral activity against influenza and antirespiratory syncytial virus which causes the lower respiratory tract infection in infants and young children in addition to other biological actions including antihepatotoxic, anti-inflammatory, antinociceptive and antioxidant effects [42].

Among the studied phytoligands classes, flavonoids showed the second most promising *in silico* activity among which chrysoeriol-7-*O*-β glucopyranoside (**12**) interacted with SARS-CoV-2-CTD Gly496 and Tyr453 through the sugar part, but not with hACE2 (Figure 3M & N). Luteolin-7-*O*-β-glucopyranoside (**14**) interacted with Tyr453, Ser494 and Gly496 at the SARS-CoV-2-CTD via the sugar hydroxyl groups; whereas, it was bound to ACE2 at His34 and Lys353 through the aromatic ring and the sugar hydroxyl group, respectively (Figure 3Q & R). Liriodendrin (Syringaresinol-di-*O*-glucoside) (**15**) exhibited hydrogen bonding with Arg403, Glu406, Arg408, Gln409 and the key Lys417 at the SARS-CoV-2-CTD side without binding to hACE2 residues (Figure 3S & T).

It is worth noting that acacetin-7-*O*-β-glucopyranoside (**13**) has been reported to exhibit anti-inflammatory action [43,44] with well-proven efficacy against chronic obstructive pulmonary disease via inhibiting neutrophilic lung inflammation in a murine model of chronic obstructive pulmonary disease [45].

Interestingly, the flavonoid, luteolin-7-*O*-β-glucopyranoside (**14**) and the lignan, liriodendrin (**15**) interacted with the key His34 and Lys353 of the hACE2 via π–π and hydrogen bonding interactions, respectively (Figure 3 O–R). It is worth mentioning that a single Lys353 mutation was reported to be sufficient to abolish the interactions at the interface. Both compounds also displayed hydrogen bonding with Tyr453, Ser494 and Gly496 of SARS-CoV-2-CTD at the interface. Benzyl alcohol-*O*-β-xylopyranosyl-(1→2)-β glucopyranoside (**4**) interacted with SARS-CoV-2-CTD Tyr453, Gly496 and Tyr505 as well as hACE2 Glu37 and Lys353 (Figure 3 E & F). Liriodendrin (**15**) was reported to exhibit protective role in sepsis-induced acute lung injury via diminishing the release of many proinflammatory mediators, *viz.* TNF-α, IL-1β, MCP-1, and IL-6 and lung myeloperoxidase accumulation in addition to suppressing the VEGF expression and NF-κB activation in the lung [46].

Among the studied iridoids (**1**, **2**, **5**–**8**), only lamiide (**6**) exhibited similar interactions with hACE2 Glu37 and Lys353 besides engaging SARS-CoV-2-CTD Glu406 and Tyr453 (Figure 3G & H). However, phlomurin (**2**) in addition to the megastigmane glucoside, phlomuroside (**3**) (Figures 3A–D) and the phenylethanoid glycoside, 2-phenylethyl-*O*-β-xylopyranosyl-(1→2)-β-glucopyranoside (**10**) (Figure 3I & J) failed to exhibit interactions with the key aminoacids of the virus–receptor complex however, they could interact with nearby residues. On the other hand, ipolamiide (**8**) and syringin (**9**) were able to fit in the binding interface without considerable interactions with its residues.

The phytoligands ‘chrysoeriol-7-*O*-β-glucopyranoside (**12**) and luteolin-7-*O*-β-glucopyranoside (**14**)' showed a slight deviation from the ideal Lipinski’s [47] and Veber’s [37] drug-like bioavailability parameters. According to Lipinski’s rule of five, phytoligands (**12** and **14**) violated the number of hydrogen bond acceptors and donors. Acteoside (11) recorded an extra Lipinski violation due to its high molecular weight. On the other hand, the studied phytochemicals violated the ideal total polar surface area of the drug-like molecule according to Veber’s parameters (number of rotatable bonds ≤10 and total polar surface area ≤140). Again, **11** showed one more violation regarding the number of rotor bonds. Pre-ADMET software [19] was employed for prediction of absorption, distribution, metabolism, excretion and toxicity (ADMET) properties of the studied natural products. **12** and **14** were predicted to display moderate human intestinal absorption. **12** recorded higher predicted absorption percentage (42%) than **14** (25%); whereas, **11** was predicted to be poorly absorbed recording only 7.6% intestinal absorption. The three phytochemicals recorded good aqueous solubility. **11** was predicted to be the most readily soluble compound among the group (S = 290.5 mg/l), followed by **14** (S = 85.91 mg/l) and **12** (S = 40.22 mg/l), respectively. This refers to the expected feasibility of the studied phytochemicals to be formulated in various pharmaceutical dosage forms. The three phytochemicals displayed comparable low CNS absorption as detected by their predicted blood–brain barrier penetration values (around 0.03). Thus, the compounds are expected to display limited possible CNS side effects. The compounds displayed medium Caco-2 model and low MDCK model permeabilities. **11** recorded the highest Caco-2 model permeability value (almost two-fold) compared with **12** and **14**. On the other hand, **14** was predicted to be two-fold more absorbable by MDCK than **12**. **11** displayed the least MDCK permeability value among the group. Interestingly, all compounds were considered poorly bound to plasma proteins (PPB ranges from 62 to 73%) indicating that much of the unbound compound will be available for transport across various membranes to display its pharmacological activities. They were predicted to be devoid of cytochromes P450 2D6 (CYP2D6) inhibition activities but not CYP3A4, addressing the possibility of limited predicted drug interactions. Finally, the median lethal dose (LD_50_; mg/kg) of the studied compounds in rodents was predicted employing ProTox [35], the toxicity predictor program, to be 5000 mg/kg; thus, classified according to the Globally Harmonized System of Classification and Labeling of Chemicals (GHS) as class V concerning acute oral toxicity.

## Conclusion

In the current study, we conducted docking simulation to predict the *in silico* inhibitory potential of a set of 15 phytoligands previously isolated *Phlomis aurea*, a wild Sinai peninsula plant, against the functional activity of SARS-CoV-Spike-ACE2 complex. Among the studied phytoligands, the phenolic glycoside ‘acteoside (**11**)’ showed the most potent *in silico* inhibitory action with an additional merit, over the reference hesperidin (**16**), of not binding to ACE2 which was reported recently to possess a pulmonary protective role in acute lung injury and acute respiratory distress syndrome *in vivo*. The second most active phytoligands were flavonoids *viz.* acacetin-7-*O*-β-glucopyranoside (**13**), chrysoeriol-7-*O*-β-glucopyranoside (**12**), followed by the lignan ‘liriodendrin (**15**)’ and the iridoid ‘lamiide (**6**)’.

Our results provide promising leads from *Phlomis aurea* plant for designing and developing drug candidates with phytoprophylactic potential against COVID-19. Besides, pulmonary inflammation and fibrosis are recognized now as the first death causes of COVID-19 patients [48].

The role of anti-inflammatory agents in the effective management of symptoms during COVID-19 has been suggested by clinical practitioners [2]. Interestingly, three of the most active phytoligands in our study have well reported anti-inflammatory actions such as acteoside (**11**), acacetin-7-*O*-β-glucopyranoside (**13**), liriodendrin (**15**), with the former compound possessed antifibrotic action additionally. Accordingly, these phytoligands could aid in developing multitargeted strategy for better management and reducing the likelihood of COVID-19 using phytomedicines. Moreover, preparation of quality controlled *P. aurea* leaf extract aiming to possess highest levels of these phytoligands is warranted. However, *in vitro* and *in vivo* experimentation now follows in order to validate the predicted antiviral potential of the most promising phytoligands in *P. aurea* and/or its leaf extract standardized to the therapeutic levels of these phytoligands. The predicted ADME and drug-likeness parameters were computed for the three most promising phytochemicals (**11**, **12** & **14**). Results showed that chrysoeriol-7-*O*-β-glucopyranoside (**12**) and luteolin-7-*O*-β-glucopyranoside (**14**) recorded relatively better predicted drug-like criteria compared with acteoside (**11**). Thus, these phytochemicals deserve to be further subjected to *in vivo* pharmacokinetic studies on experimental animals.

**Summary points**

- *In silico* analysis of 15 *Phlomis aurea* phytoligands against SARS-CoV-Spike-ACE2 complex.
- Acteoside possessed the most potent *in silico* inhibition without binding to ACE2.
- The study posed some phytoligands with prophylactic potential against COVID-19.
- 7-*O*-β-glucopyranosides of chrysoeriol and luteolin possessed optimum drug-like criteria.
